# Psychological and Psychiatric Characteristics of People with Keratoconus

**DOI:** 10.3390/reports7030067

**Published:** 2024-08-03

**Authors:** Szymon Florek, Piotr Gościniewicz, Magdalena Suszka, Ewa Mrukwa-Kominek, Robert Pudlo

**Affiliations:** 1 Doctoral School, Department of Psychoprophylaxis, Faculty of Medical Sciences in Zabrze, Medical University of Silesia, 40-055 Katowice, Poland; 2 Faculty of Medicine, WSB University, 41-300 Dąbrowa Górnicza, Poland; piotr.gosciniewicz@gmail.com; 3 Clinical Department of Psychiatry, Multi-Specialist District Hospital, 42-612 Tarnowskie Góry, Poland; 4 Department of Ophthalmology, Faculty of Medical Sciences in Katowice, Medical University of Silesia, 40-055 Katowice, Poland; ewa.mrukwa@sum.edu.pl; 5 Department of Psychoprophylaxis, Faculty of Medical Sciences in Zabrze, Medical University of Silesia, 40-055 Katowice, Poland; rpudlo@sum.edu.pl

**Keywords:** keratoconus, personality, depression, anxiety, ophthalmology

## Abstract

*Background and Objectives*: There are many reports in the literature on the co-occurrence of somatic diseases and psychiatric disorders. Relatively few have addressed the co-occurrence of corneal cone with anxiety, depression, or personality disorders. *Materials and Methods*: 99 patients with keratoconus (study group) and 92 patients without keratoconus (control group) participated in the entire study, which was conducted in 2015 and 2020–2023. The Hamilton Depression Rating Scale (HDRS) and Beck’s Depression Inventory (BDI) were used to assess depressive symptoms, the State-Trait Anxiety Inventory (STAI) to assess anxiety symptoms, and the DSM-IV Personality Disorder Inventory (IBZO-DSM-IV) to identify personality disorders. *Results*: In the study group, the severity of depressive and anxiety symptoms correlated with all types of personality disorders. In the control group, these symptoms did not correlate with antisocial and narcissistic personalities. In the comparative analysis, there were more patients with antisocial, schizotypal, obsessive compulsive, schizoid, paranoid, and dependent personality traits in the study group. *Conclusions*: Patients with keratoconus have increased expressions of antisocial personality traits, but no differences in anxiety and depression symptoms were evident. Further research is needed among patients diagnosed with keratoconus in the short term.

## 1. Introduction

The links between mental state and somatic health have been widely studied. In relation to these, concepts such as psychosomatic disorders have been created [1], and the search for the origins of mental disorders in, for example, the gut microbiota has begun [2]. There are also reports in the literature on the co-occurrence of psychiatric disorders and eye diseases [3]. A topic equally vast is the impact of somatic diseases on mental health. The impact of chronic diseases such as hypertension, diabetes, and chronic kidney disease on patients’ mental health has been extensively studied [4,5,6].

Evaluating the impact of somatic diseases on mental state can be difficult due to its conditioning by various factors. In addition, a reliable study of mental state should be neutral, measurable, and repeatable. For this reason, a variety of scales have been developed with which to determine the intensity of anxiety and the presence of factors that may indicate a depressive episode or personality disorders [7,8,9,10].

Keratoconus is an ophthalmic condition in which progressive visual disturbances occur. They are caused by progressive ectasia and thinning of the cornea, which results in irregular astigmatism. Corneal stippling affects the central or paracentral cornea and is most often located inferiorly. There are several theories regarding the pathogenesis of the keratoconus, but none of them have been confirmed [11]. Until now, keratoconus has been described as a non-inflammatory disease [12,13], but several studies have reported an association of the presence of keratoconus with significant changes in inflammatory mediators [14,15,16,17], indicating that some form of inflammation is present [18,19,20]. Although it is usually a bilateral condition, one eye is usually more affected by corneal ectasia than the other. Regarding the prevalence of keratoconus, studies are also inconclusive and suggest considerable geographic–ethnic variation. The prevalence is estimated between 0.2 and 4790 patients per 100,000 people, and the disease is most commonly diagnosed in young adults, with an average age of 28.3 years [21,22]. The question of how the incidence of keratoconus varies between the sexes also seems unclear, as reports of no difference, a higher incidence in women, and a higher incidence in men can all be found, depending on the study [23].

In the presence of a progressive disease which gradually deteriorates vision, the potential development of psychiatric disorders should be considered. It seems reasonable to assess the severity of anxiety and depression, since, as other studies indicate, they may be more common in people with somatic diseases than in the general population [24,25]. Nevertheless, due to the young age of onset of keratoconus, special attention should be paid to the issue of the presence of potential personality disorders. As studies have shown, the abnormal formation of personality structure in adolescents can result in impaired consistent functioning in adulthood [26]. Therefore, research analysing the personality of patients with keratoconus is needed.

The purpose of the present study was to determine the links between the presence of keratoconus and anxiety disorders, depressive disorders, and personality disorders.

For the present study, an exploratory hypothesis was developed that keratoconus patients are over-represented with personality disorders. This was based on an analysis of the available literature on the subject. However, due to the minimal number of reliable studies assessing more mental health parameters in keratoconus patients, it was decided to extend the present project to assess the intensity of anxiety and depression symptoms.

## 2. Materials and Methods

After approval from the Bioethics Committee (resolution no. BNW/NWN/0052/KB1/24/23) operating at the Silesian Medical University, a study was conducted among patients of the Eye Refractive Surgery Clinic operating at the Clinical Department of Adult Ophthalmology at the Prof. K. Gibinski University Clinical Center in Katowice. This centre was chosen because of the large number of patients with keratoconus. Each survey participant gave written consent before taking the survey. Respondents were informed that the survey was a single-item survey and posed no risk to the life and health of the participants. They were also informed that they could opt out of the survey at any time. The survey, for reasons of organisational and technical constraints, was conducted in two time periods—the first 20 people in the study population took part in the survey in 2015, while further people were recruited between 2020 and 2023. There were none of the same participants in these two different time periods. Moreover, they received the same medical care. The project involved testing approximately 100 people with a diagnosis of corneal cone (study group) and another approximately 100 people without this diagnosis (control group). The inclusion criterium common to the study and control group was an age of 18 years. Informed consent to participate in the study was a prerequisite for each study participant. In addition, for the study group, another inclusion criterion was the diagnosis of keratoconus, while, for the control group, its absence was necessary. Exclusion criteria common to both groups were cognitive impairment making it difficult or impossible to complete the questionnaires, as well as a previous or current diagnosis of intellectual disability (F70-F79), dementia and other organic disorders (F00-F09), schizophrenia and related disorders (F20-F29), or bipolar disorder (F30, F31). The control group consisted of declaratively healthy people without keratoconus, but, nevertheless, their health status was not controlled by any tests.

The diagnosis of keratoconus was carried out by an ophthalmologist specialist with many years of experience, using a videokeratograph. Patients with a confirmed diagnosis of keratoconus at a minimum of three visits to the Eye Refraction Clinic and no progression were eligible for the study. Based on the examination of the corneal topography, the degree, type, and shape of the corneal cone were determined. Classification of the keratoconus was carried out based on the analysis of the mean value of the central rays. The Amsler and Krumeich scale was used for classification. This classification is based on measurements of mean K values on sagittal maps of anterior corneal curvature, pachymetry values at the thinnest point of the cornea, and the patient’s refractive defect [27,28,29]. The corneal surface was assessed using images obtained in Casia 2 Anterior Segment OCT, Tomey, Japan. Keratoconus tests were performed using the keratoconus probability programme in the Topography Modeling System TMS4, Tomey, Japan.

Before entering the study, each participant was thoroughly briefed on the processing of personal data and the rules for taking part in the study. The subjects were informed that they could stop the study at any time without consequences for their health and further diagnostic and therapeutic process. After written consent was obtained in duplicate, one of which remained with the subject and the other with the researcher, the actual study proceeded.

Study participants were surveyed with a set of standardised psychometric tools to identify potential mental health disorders. The examination took place in a separate doctor’s room after the ophthalmology appointments. Patients were given privacy during the examination, the environment was quiet, and there was no limit to the length of the examination—it took as long as the patient required. The examination was supervised by a clinical psychology specialist.

### 2.1. Hamilton Depression Rating Scale (HDRS)

This is a scale completed by the examiner. It consists of 17 items presenting various symptoms of depression. The examiner assesses these symptoms with 9 points on a five-point scale and another 8 points (including one with two subscores) on a three-point scale. The total score is obtained by simply adding up the achieved points, and the maximum score is 54 points [30]. In our project, we used a Polish adaptation of HDRS [31].

### 2.2. Beck’s Depression Inventory (BDI)

This is a commonly used self-report questionnaire for screening for depressive disorders. It consists of 21 statements to which the respondent should respond according to a four-point Likert scale. The total score is obtained by a simple sum of the scores obtained, and the maximum score is 63 points [32,33]. The study used the Polish validation of the tool described [31]. It was decided to use two scales to describe the intensity of depressive symptoms, utilizing their different natures. While the HDRS examines the possibly objective symptoms of depression, the BDI is a scale measuring the subjective symptoms.

### 2.3. State-Trait Anxiety Inventory (STAI)

This is a commonly used inventory for anxiety disorders. It consists entirely of 40 statements that the respondent must answer on a 4-point Likert scale. The inventory has two subscales—one measures anxiety as a personality trait, while the other measures anxiety as a state at the time of the survey. The scores obtained are subject to the sum, but reverse scoring applies to some of the questions [34]. In this study,, a Polish validation of the scale was used [35].

### 2.4. Inventory for the Study of Personality Disorders According to DSM-IV (IBZO-DSM-IV)

The inventory was developed by Radochonski and Stanik and is used to identify ten different types of personality disorders. The entire inventory consists of 100 statements, and the respondent is asked to respond to them using a 3-point scale, where 0 means that the statement does not fit the respondent, and 2 means that it accurately characterises them. It is recommended that as few statements as possible are marked as 1, which means that the given statement “somewhat characterizes the respondent”. Scores are obtained by summing the points separately for each type of personality disorder. The resulting scores were converted to a 10-point scale to normalise them and allow for later analysis [36].

### 2.5. Statistical Analysis

Statistical analysis was carried out using Excel 365 and Statistica 13.3 software. To assess the normality of the results’ distribution, an analysis was performed using the Shapiro–Wilk W test. Then, correlation analyses were performed using Spearman’s rank correlation. In comparative analyses, Mann–Whitney U tests and chi-squared tests were used for variables converted according to accepted psychometric norms. All statistical considerations presented were conducted at the α = 0.05 level of significance.

## 3. Results

### 3.1. Descriptive Analysis

A total of 99 patients with a diagnosis of corneal cone were included in the project, of whom 26 (26.26%) were women and 73 (73.73%) were men. The mean age in the study group was 31.39 ± 11.30 years. The ongoing project also recruited a control group of 92 people, of which 38 (41.30%) were women and 54 (58.70%) were men. The mean age in the control group was 28.77 ± 9.26 years. Due to the rather large overrepresentation of men in the study group, there were difficulties when adequately matching the control group, as discussed in detail in the section outlining the limitations of the paper. A full summary of the descriptive statistics, together with the results of the Shapiro–Wilk test, is included in Supplementary Table S1.

### 3.2. Correlational Analysis

A correlational analysis within the study population showed moderately strong positive correlations of depression scale scores with all personality disorders studied. However, only BDI scores correlated positively with both STAI subscales, and HDRS scores correlated positively only with the anxiety-as-trait subscale (STAI X-1). A detailed analysis of the correlations in the study group is shown in Table 1.

Appropriate correlational analyses were also performed with the study group by gender, but as a thorough analysis of these is beyond the scope of this article—their results are presented in the Supplementary Material, with Table S2 detailing the women’s results and Table S3 detailing the men’s results, respectively.

Adequate correlation analyses were performed for the control group and the results are presented in Table 2.

The results of the correlation analyses of the study variables within the control group by gender are presented in Table S4 for women and Table S5 for men, respectively.

### 3.3. Comparisons Analysis

The study variables were compared between the test and control groups, obtaining, for raw scores (BDI, HDRS) and standard ten (Sten) scores (STAI, IBZO-DSM-IV), statistically significant differences in the severity of schizotypal and antisocial personality traits, as shown in Table 3.

Similar analyses were performed with the gender breakdown. Among women, differences were identified in the expression of schizotypal and obsessive compulsive personality traits, as shown in Table 4. In the male group, no statistically significant differences were identified, as shown in Supplementary Table S6.

For the study variables, comparisons were also made between the test and control groups after recalculating the scale scores according to recognised norms. For the IBZO-DSM-IV scales, a cut-off point of 7 Sten points was taken as the limit of elevated expression of the traits in question. The analysis of the recalculated results from the HDRS, BDI, and STAI scales was not performed due to a lack of statistical significance. The obtained results from the IBZO-DSM-IV scale are presented in Table 5.

Adequate analyses in terms of recalculated IBZO-DSM-IV scale scores were performed separately for women and men, as shown in Table 6 and Table 7, respectively.

No statistically significant differences were evident in the analyses of the converted scores of the other scales, as shown in the corresponding Tables S7–S9.

## 4. Discussion

The difference between the control and study groups only includes the lack of correlation of depressive symptom severity with the expression of antisocial and narcissistic personality traits in the former (Table 2). However, these results are not surprising in relation to previous scientific reports [37,38].

In contrast, the correlations of anxiety with the expression of traits of individual personality disorders appear to be universal and independent of keratoconus disease. When comparing the correlations in the study and control groups, no large difference can be visualised, which is also confirmed by the analyses presented in the Supplementary Materials. The prevalence of positive correlations of anxiety intensity with personality disorders is confirmed by a meta-analysis conducted by Norwegian researchers [39].

The single studies conducted to date on anxiety intensity and depression among people with keratoconus are inconclusive, as discussed in detail in a separate article [40]. In the project conducted by the authors, no differences in this regard between the study and control group were highlighted (Table 3).

Finally, it is necessary to turn to the analysis of the comparisons presented on the expression of the different personality types’ traits. As mentioned, a higher percentage of individuals with antisocial personality traits in the study group is noticeable in most of the analyses. In addition, when comparing directly the results obtained from the scales, one should notice a higher expression of schizotypal traits in the entire study population and among the female respondents. Importantly, this difference is not statistically significant after taking the limit of seven Sten scores as the limit of pathological expression of the mentioned traits. This suggests that the keratoconus patients studied presented more schizotypal traits, but that these traits did not reach an intensity that met the criteria for personality disorders. The expression of obsessive compulsive traits is quite different in women, which is evident both in the comparison of Sten scores directly and after adopting the aforementioned division (Table 4 and Table 6). Moreover, when the results are recalculated according to the adopted norms, statistically significant differences are apparent in the higher percentage of patients in the study group who struggle with schizoid, paranoid, and dependent disorders (Table 5, Table 6 and Table 7). The results obtained in terms of personality disorders are in line with previous studies on people with keratoconus [41,42,43,44,45]. The increased prevalence of obsessive compulsive personality among the female subjects, although not shown in a study conducted in 1987, confirms the findings of a more recent one published by Aslan et al. in 2021 [44,45]. The multiplicity and diversity of personality disorders potentially present in patients with keratoconus may be indicative of a personality pattern that breaks out of the accepted division. Given the previously mentioned studies, it seems reasonable to take the traits described in them—passive aggression, neuroticism, a lack of respect, paranoia, and hypomania—as those that accurately characterise people with keratoconus. It is important to note that in our study, these traits may have accurately described the participants in the study group. Which traits were more prominent in a particular patient determined the final score for a specific personality disorder.

When considering personality disorders in relation to chronic and progressive somatic illness, an analysis of previous research should also be conducted. At this point, it should be noted that Kraepelin had already created the concept of homilopathies—psychiatric disorders categorised as so-called Verkerspsychosen, psychoses that are a reaction to the impaired perception of surrounding phenomena. Moreover, homilopathies could appear in people who are unable to interact with their surroundings due to chronic illness [46]. Nevertheless, the concept seems to have been forgotten, which does not exclude the continued existence of the described mechanisms and research on the impact of chronic illnesses on the psyche. One study indicates that children and adolescents with chronic illnesses are at risk of delays in social and psychosexual development [47]. Another focuses on the level of self-esteem highlighting its significant reduction in young adults with chronic illness compared to healthy adults [5]. Previous research emphasises the changes in patients with chronic diseases in terms of a decrease in extraversion, conscientiousness, and openness to new experiences, as well as a weakening of emotional stability [5,6]. Analysing the results of our study, it can be seen that some features of antisocial personality, but also schizoid, schizotypal, anankastic, paranoid, and dependent personality are part of the disorders in question. They could be the result of chronic illnesses present during adolescence. It seems that, to properly define potential psychiatric disorders, and even more so to effectively prevent them, it is crucial to conduct an adequate study using separate scales among adolescents with a recent diagnosis of keratoconus. Currently, a draft of this project from the same team is being reviewed by the Bioethics Committee. Such a study will help to determine whether the disorder presented is the result of a previous depressive experience, which would be associated with the possibility of preventing the development of later disorders by the early inclusion of antidepressant therapy, or whether it is unrelated.

### Limitations and Strengths

In the present study, it was possible to collect groups with relatively large size. It should be mentioned that this study was carried out in the largest ophthalmology centre in Silesia, where patients with this condition are treated. Furthermore, when comparing the number of study subjects in the present project to other reports published to date, only three of them had a higher number of patients with keratoconus [41,43,48]. The present study was conducted with a control group of comparable size, which strongly distinguishes it from others in which there was no control group at all or a significant disparity in size between the study and control groups [6,41,43,44,49]. The interpretation of the results may be hampered by the disproportion between men and women. The difficulty of matching a suitable control group of predominantly young men seems obvious. Compared to previous reports, this study took into account the largest number of potential mental health variables—in addition to focusing on personality disorders, the expression of anxiety and depression was also determined. In addition, an important clinical implication of the study is the need to pay attention to the psychological state of patients with keratoconus, as neglecting the psychiatric and psychological aspects of treatment results in poorer quality of life and cooperation [50].

## 5. Conclusions

1. Patients with keratoconus most commonly present an increased intensity of antisocial personality traits.
2. The relationship between the severity of depression, anxiety symptoms, and the personality disorder is not disrupted in people with keratoconus.
3. The diagnosis of keratoconus does not affect the severity of depressive and anxiety symptoms.

## Figures and Tables

**Table 1:** Spearman rank correlations within the study population (Spearman’s rank, *p* < 0.05).

Variable	HDRS	BDI	STAI X-1	STAI X-2
HDR	1.000	0.531 *	0.344 *	0.447 *
BDI		1.000	0.458 *	0.613 *
STAI X-1			1.000	0.624 *
STAI X-2				1.000
paranoid personality	0.430 *	0.626 *	0.524 *	0.567 *
schizoid personality	0.406 *	0.434 *	0.456 *	0.463 *
schizotypal personality	0.439 *	0.682 *	0.448 *	0.493 *
antisocial personality	0.359 *	0.461 *	0.227	0.224
borderline personality	0.462 *	0.650 *	0.454 *	0.665 *
histrionic personality	0.331 *	0.537 *	0.229 *	0.433 *
narcissistic personality	0.396 *	0.471 *	0.190	0.395 *
avoidant personality	0.389 *	0.591 *	0.357 *	0.624 *
dependent personality	0.349 *	0.502 *	0.438 *	0.654 *
obsessive compulsive personality	0.266 *	0.488 *	0.225	0.370 *

*—statistically significant at *p* < 0.05; HDRS—Hamilton Depressing Rating Scale; BDI—Beck’s Depression Inventory; STAI X-1—State-Trait Anxiety Inventory for anxiety as a condition [ten]; STAI X-2—State-Trait Anxiety Inventory for anxiety as a trait [ten].

**Table 2:** Spearman rank correlations within the control group (Spearman’s rank, *p* < 0.05).

Variable	HDRS	BDI	STAI X-1	STAI X-2
HDR	1.000	0.701 *	0.591 *	0.605 *
BDI		1.000	0.677 *	0.713 *
STAI X-1			1.000	0.956 *
STAI X-2				1.000
paranoid personality	0.466 *	0.579 *	0.448 *	0.450 *
schizoid personality	0.267 *	0.525 *	0.486 *	0.412 *
schizotypal personality	0.435 *	0.549 *	0.334 *	0.438 *
antisocial personality	0.020	−0.080	−0.123	−0.186
borderline personality	0.577 *	0.662 *	0.568 *	0.654 *
histrionic personality	0.241 *	0.337 *	0.141	0.236 *
narcissistic personality	0.095	0.188	0.112	0.182
avoidant personality	0.348 *	0.531 *	0.382 *	0.623 *
dependent personality	0.398 *	0.509 *	0.342 *	0.533 *
obsessive compulsive personality	0.246 *	0.306 *	0.287	0.336 *

*—statistically significant at *p* < 0.05; HDRS—Hamilton Depressing Rating Scale; BDI—Beck’s Depression Inventory; STAI X-1—State-Trait Anxiety Inventory for anxiety as a condition [ten]; STAI X-2—State-Trait Anxiety Inventory for anxiety as a trait [ten].

**Table 3:** Comparison of study parameters between test and control group (Mann–Whitney U test, *p* < 0.05).

Variable	Study Group (*n* = 99)			Control Group (*n* = 92)			Cohen’s d	Z	*p*
	Average	SD	Median	Average	SD	Median			
HDR	3.586	4.145	2.000	3.478	4.775	1.500	0.116	0.800	0.424
BDI	5.727	6.882	4.000	5.967	6.336	4.000	0.068	−0.466	0.641
STAI X-1	4.840	1.987	5.000	5.120	2.132	5.000	0.125	−0.803	0.422
STAI X-2	4.213	2.297	4.000	4.598	2.423	4.000	0.160	−1.031	0.302
paranoid personality	4.475	2.422	4.000	3.859	1.976	4.000	0.227	1.555	0.120
schizoid personality	4.765	2.465	4.000	4.870	1.985	5.000	0.162	−1.004	0.315
schizotypal personality	4.747	2.463	4.000	3.957	2.054	4.000	0.327	2.228	<0.05 *
antisocial personality	4.434	2.286	4.000	3.598	1.816	4.000	0.325	2.216	<0.05 *
borderline personality	4.040	2.445	4.000	3.989	2.265	4.000	0.005	0.035	0.972
histrionic personality	4.434	2.417	4.000	4.315	1.950	4.000	0.026	0.177	0.860
narcissistic personality	4.000	2.433	4.000	4.435	1.957	4.000	0.202	−1.386	0.166
avoidant personality	4.828	2.356	4.000	4.946	2.206	5.000	0.035	−0.241	0.810
dependent personality	5.293	2.467	5.000	5.011	2.083	5.000	0.086	0.591	0.555
obsessive compulsive personality	5.020	2.245	5.000	4.641	2.241	5.000	0.175	1.205	0.228

*—statistically significant at *p* < 0.05; HDR—Hamilton Depressing Rating; BDI—Beck’s Depression Inventory; STAI X-1—State-Trait Anxiety Inventory for anxiety as a condition [ten]; STAI X-2—State-Trait Anxiety Inventory for anxiety as a trait [ten]; SD—standard deviation.

**Table 4:** Comparison of study parameters among women between test and control group (Mann–Whitney U test, *p* < 0.05).

Variable	Study Group (*n* = 26)			Control Group (*n* = 38)			Cohen’s d	Z	*p*
	Average	SD	Median	Average	SD	Median			
HDR	4.808	5.114	3.500	4.316	5.705	2.500	0.257	1.012	0.312
BDI	8.000	8.532	6.000	7.026	6.824	5.500	0.062	0.239	0.811
STAI X-1	5.864	1.859	6.000	5.368	2.123	5.000	0.491	0.966	0.334
STAI X-2	4.682	2.079	4.000	4.579	2.226	5.000	0.274	0.046	0.963
paranoid personality	5.038	2.323	5.000	3.947	2.277	4.000	0.498	1.927	0.054
schizoid personality	5.654	2.607	5.000	4.789	1.711	5.000	0.218	0.861	0.389
schizotypal personality	5.615	2.351	5.000	3.816	2.065	4.000	0.750	2.802	<0.001 *
antisocial personality	4.154	2.167	4.000	3.263	1.811	3.000	0.361	1.415	0.157
borderline personality	4.500	2.045	4.000	4.237	2.454	4.000	0.156	0.615	0.538
histrionic personality	4.154	2.222	4.000	4.053	2.039	4.000	0.068	0.267	0.790
narcissistic personality	3.962	2.010	4.000	3.868	1.803	5.000	0.101	0.396	0.692
avoidant personality	4.962	2.457	4.500	4.921	2.352	5.000	0.021	0.075	0.940
dependent personality	5.385	2.192	5.000	4.789	2.407	4.500	0.345	1.353	0.176
obsessive compulsive personality	5.308	2.259	5.000	4.158	2.060	3.500	0.536	2.064	<0.05 *

*—statistically significant at *p* < 0.05; HDR—Hamilton Depressing Rating; BDI—Beck’s Depression Inventory; STAI X-1—State-Trait Anxiety Inventory for anxiety as a condition [ten]; STAI X-2—State-Trait Anxiety Inventory for anxiety as a trait [ten]; SD—standard deviation.

**Table 5:** Comparison of IBZO-DSM-IV scale scores recalculated according to the adopted Sten norms (chi-squared test, *p* < 0.05).

Variable	Study Group (*n* = 99)		Control Group (*n* = 92)		Chi-Squared	*p*
	*N*	Percentage [%]	*N*	Percentage [%]		
paranoid personality [ten]					4.809	<0.05 *
<7	75	75.76	81	88.04		
≥7	24	24.24	11	11.96		
schizoid personality [ten]					0.446	0.504
<7	78	78.79	76	82.61		
≥7	21	21.21	16	17.39		
schizotypal personality [ten]					2.746	0.098
<7	77	77.78	80	86.96		
≥7	22	22.22	12	13.04		
antisocial personality [ten]					8.955	<0.01 *
<7	81	81.82	88	95.65		
≥7	18	18.18	4	4.35		
borderline personality [ten]					0.695	0.405
<7	83	83.84	81	88.04		
≥7	16	16.16	11	11.96		
histrionic personality [ten]					0.414	0.520
<7	84	84.85	81	88.04		
≥7	15	15.15	11	11.96		
narcissistic personality [ten]					0.371	0.543
<7	83	83.84	80	86.96		
≥7	16	16.16	12	13.04		
avoidant personality [ten]					1.748	0.186
<7	74	74.75	76	82.61		
≥7	25	25.25	16	17.39		
dependent personality [ten]					4.348	<0.05 *
<7	69	69.70	76	82.61		
≥7	30	30.30	16	17.39		
obsessive compulsive personality [ten]					3.751	0.053
<7	70	70.71	76	82.61		
≥7	29	29.29	16	17.39		

*—statistically significant at *p* < 0.05; *N*—number of people.

**Table 6:** Comparison of IBZO-DSM-IV scale scores recalculated according to the adopted Sten norms among women (chi-squared test, *p* < 0.05).

Variable	Study Group (*n* = 26)		Control Group (*n* = 38)		Chi-Squared	*p*
	*N*	Percentage [%]	*N*	Percentage [%]		
paranoid personality [ten]					0.207	0.649
<7	20	76.92	31	81.58		
≥7	6	23.08	7	18.42		
schizoid personality [ten]					4.159	<0.05 *
<7	17	65.38	33	86.84		
≥7	19	34.62	5	13,16		
schizotypal personality [ten]					2.958	0.085
<7	18	69.23	33	86.84		
≥7	8	30.77	5	13,16		
antisocial personality [ten]					5.006	<0.05 *
<7	21	80.77	37	97.37		
≥7	5	19.23	1	02.63		
borderline personality [ten]					0.555	0.456
<7	23	88.46	31	81.58		
≥7	3	11.54	7	18.42		
histrionic personality [ten]					0.001	0.976
<7	24	92.31	35	92.11		
≥7	2	07.69	3	07.89		
narcissistic personality [ten]					0.001	0.976
<7	24	92.31	35	92.11		
≥7	2	07.69	3	07.89		
avoidant personality [ten]					0.538	0.463
<7	20	76.92	32	84.21		
≥7	6	23.08	6	15.79		
dependent personality [ten]					0.653	0.419
<7	19	73.08	31	81.58		
≥7	7	26.92	7	18.42		
obsessive compulsive personality [ten]					4.232	<0.05 *
<7	16	61.54	32	84.21		
≥7	10	38.46	6	15.79		

*—statistically significant at *p* < 0.05; *N*—number of people.

**Table 7:** Comparison of IBZO-DSM-IV scale scores recalculated according to the adopted Sten norms among men (chi-squared test, *p* < 0.05).

Variable	Study Group (*n* = 73)		Control Group (*n* = 54)		Chi-Squared	*p*
	*N*	Percentage [%]	*N*	Percentage [%]		
paranoid personality [ten]					6.449	<0.05 *
<7	55	75.34	50	92.59		
≥7	18	24.66	4	07.41		
schizoid personality [ten]					0.324	0.569
<7	61	83.56	43	79.63		
≥7	12	16.44	11	20.37		
schizotypal personality [ten]					0.869	0.351
<7	59	80.82	47	87.04		
≥7	14	19.18	7	12.96		
antisocial personality [ten]					4.232	<0.05 *
<7	60	82.19	51	94.44		
≥7	13	17.81	3	05.56		
borderline personality [ten]					2.896	0.089
<7	60	82.19	50	92.59		
≥7	13	17.81	4	07.41		
histrionic personality [ten]					0.202	0.653
<7	60	82.19	46	85.19		
≥7	13	17.81	8	14.81		
narcissistic personality [ten]					0.132	0.716
<7	59	80.82	45	83.33		
≥7	14	19.18	9	16.67		
avoidant personality [ten]					0.993	0.319
<7	54	73.97	44	81.48		
≥7	19	26.03	10	18.52		
dependent personality [ten]					3.627	0.057
<7	50	68.49	45	83.33		
≥7	23	31.51	9	16.67		
obsessive compulsive personality [ten]					0.993	0.319
<7	54	73.97	44	81.48		
≥7	19	26.03	10	18.52		

*—statistically significant at *p* < 0.05; *N*—number of people.

## Data Availability

The raw data supporting the conclusions of this article will be made available by the authors on request.

## Supplementary Materials

The following supporting information can be downloaded at: https://www.mdpi.com/article/10.3390/reports7030067/s1, Table S1. Descriptive statistics and results of Shapiro-Wilk tests among analysed variables; Table S2: Spearman rank correlations within the women of the study population. Table S3: Spearman rank correlations within the men of the study population. Table S4: Spearman rank correlations within the women of the control group. Table S5: Spearman rank correlations within the men of the control group. Table S6: Comparison of study parameters among men between test and control group (Mann–Whitney U test, *p* < 0.05). Table S7: Comparative analysis of the severity of depressive and anxiety symptoms in the test and control group (chi-squared test, *p* < 0.05). Table S8: Comparative analysis of the severity of depressive and anxiety symptoms among women in the test and control group (chi-squared test, *p* < 0.05). Table S9: Comparative analysis of the severity of depressive and anxiety symptoms among men in the test and control group (chi-squared test, *p* < 0.05).

## References

[1] Chandrashekar C.R., Math S.B. (2006). Psychosomatic disorders in developing countries: Current issues and future challenges. Curr. Opin. Psychiatry.

[2] Mörkl S., Butler M.I., Holl A., Cryan J.F., Dinan T.G. (2020). Probiotics and the Microbiota-Gut-Brain Axis: Focus on Psychiatry. Curr. Nutr. Rep..

[3] Sadykov E., Studnička J., Hosák L., Siligardou M.R., Elfurjani H., Hoikam J.L., Kugananthan S., Petrovas A., Amjad T. (2019). The Interface between Psychiatry and Ophthalmology. Acta Medica.

[4] Voorend C.G., van Buren M., Berkhout-Byrne N.C., Kerckhoffs A.P., van Oevelen M., Gussekloo J., Richard E., Jan WBos W., Mooijaart S.P. (2024). Apathy Symptoms, Physical and Cognitive Function, Health-Related Quality of Life, and Mortality in Older Patients with CKD: A Longitudinal Observational Study. Am. J. Kidney Dis..

[5] Liu S.Y., Wrosch C., Morin A.J.S., Quesnel-Vallée A., Pruessner J.C. (2019). Changes in self-esteem and chronic disease across adulthood: A 16-year longitudinal analysis. Soc. Sci. Med..

[6] Jokela M., Hakulinen C., Singh-Manoux A., Kivimäki M. (2014). Personality change associated with chronic diseases: Pooled analysis of four prospective cohort studies. Psychol. Med..

[7] Julian L.J. (2011). Measures of anxiety: State-Trait Anxiety Inventory (STAI), Beck Anxiety Inventory (BAI), and Hospital Anxiety and Depression Scale-Anxiety (HADS-A). Arthritis Care Res..

[8] Uher R., Farmer A., Maier W., Rietschel M., Hauser J., Marusic A., Mors O., Elkin A., Williamson R.J., Schmael C. (2008). Measuring depression: Comparison and integration of three scales in the GENDEP study. Psychol. Med..

[9] Sellbom M., Brown T.A., Bagby R.M. (2020). Validation of MMPI-2-RF Personality Disorder Spectra scales in a psychiatric sample. Psychol. Assess..

[10] Loo R. (1979). A psychometric investigation of the Eysenck Personality Questionnaire. J. Pers. Assess..

[11] Davidson A.E., Hayes S., Hardcastle A.J., Tuft S.J. (2014). The pathogenesis of keratoconus. Eye.

[12] Rabinowitz Y.S. (1998). Keratoconus. Surv. Ophthalmol..

[13] Krachmer J.H., Feder R.S., Belin M.W. (1984). Keratoconus and related noninflammatory corneal thinning disorders. Surv. Ophthalmol..

[14] Wisse R.P.L., Kuiper J.J.W., Gans R., Imhof S., Radstake T.R.D.J., Van Der Lelij A. (2015). Cytokine expression in keratoconus and its corneal microenvironment: A systematic review. Ocul. Surf..

[15] Jun A.S., Cope L., Speck C., Feng X., Lee S., Meng H., Hamad A., Chakravarti S. (2011). Subnormal cytokine profile in the tear fluid of keratoconus patients. PLoS ONE.

[16] Lema I., Durán J.A. (2005). Inflammatory molecules in the tears of patients with keratoconus. Ophthalmology.

[17] Lema I., Sobrino T., Durán J.A., Brea D., Díez-Feijoo E. (2009). Subclinical keratoconus and inflammatory molecules from tears. Br. J. Ophthalmol..

[18] Balasubramanian S.A., Mohan S., Pye D.C., Willcox M.D.P. (2012). Proteases, proteolysis and inflammatory molecules in the tears of people with keratoconus. Acta Ophthalmol..

[19] Galvis V., Sherwin T., Tello A., Merayo J., Barrera R., Acera A. (2015). Keratoconus: An inflammatory disorder?. Eye.

[20] McMonnies C.W. (2015). Inflammation and keratoconus. Optom. Vis. Sci..

[21] Santodomingo-Rubido J., Carracedo G., Suzaki A., Villa-Collar C., Vincent S.J., Wolffsohn J.S. (2022). Keratoconus: An updated review. Cont. Lens Anterior Eye.

[22] Godefrooij D.A., de Wit G.A., Uiterwaal C.S., Imhof S.M., Wisse R.P. (2017). Age-specific Incidence and Prevalence of Keratoconus: A Nationwide Registration Study. Am. J. Ophthalmol..

[23] Romero-Jiménez M., Santodomingo-Rubido J., Wolffsohn J.S. (2010). Keratoconus: A review. Cont. Lens Anterior Eye.

[24] Henning M., Subic-Wrana C., Wiltink J., Beutel M. (2020). Anxiety Disorders in Patients with Somatic Diseases. Psychosom. Med..

[25] Holt R.I., de Groot M., Golden S.H. (2014). Diabetes and depression. Curr. Diab Rep..

[26] Lenkiewicz K., Srebnicki T., Bryńska A. (2016). Mechanisms shaping the development of personality and personality disorders in children and adolescents. Psychiatr. Pol..

[27] Panthier C., Moran S., Bourges J.L. (2020). Evaluation of vision-related quality of life in keratoconus patients, and associated impact of keratoconus severity indicators. Graefes Arch. Clin. Exp. Ophthalmol..

[28] Berdasco F., Muñoz A., Fernández C., González S., Arbaiza B. (2023). Clinical-epidemiological characteristics of keratoconus in Asturias. Arch. Soc. Esp. Oftalmol..

[29] Avetisov S.E., Averich V.V., Pateyuk L.S. (2023). Keratoconus: Main lines of research. Vestn. Oftalmol..

[30] Hamilton M. (1960). A rating scale for depression. J. Neurol. Neurosurg. Psychiatry.

[31] Rybakowski J., Pużyński S., Wciórka J. (2010). Psychiatria. Podstawy Psychiatrii.

[32] Beck A.T., Ward C.H., Mendelson M., Mock J., Erbaugh J. (1961). An inventory for measuring depression. Arch. Gen. Psychiatry.

[33] Beck A.T., Steer R.A., Garbin M.G. (1988). Psychometric properties of the Beck Depression Inventory: Twenty-five years of evaluation. Clin. Psychol. Rev..

[34] Spielberger C.D. (1989). State-Trait Anxiety Inventory: Bibliography.

[35] Wrześniewski K., Sosnowski T., Jaworowska A., Fecenec D. (2011). Inwentarz Stanu i Cechy Lęku STAI: Polska Adaptacja STAI: Podręcznik.

[36] Stanik J.M. (2006). Inwentarz do Badania Zaburzeń Osobowości Według DSM-IV.

[37] Valerio M.P., Blasco B., Tagni F., Szmulewicz A.G., Martino D.J. (2020). Personality Disturbances in Melancholic and Nonmelancholic Unipolar Major Depression: A Systematic Review and Meta-Analysis. J. Nerv. Ment. Dis..

[38] Fava M., Farabaugh A.H., Sickinger A.H., Wright E., Alpert J.E., Sonawalla S., Nierenberg A.A., Worthington J.J. (2002). Personality disorders and depression. Psychol. Med..

[39] Friborg O., Martinussen M., Kaiser S., Overgård K.T., Rosenvinge J.H. (2013). Comorbidity of personality disorders in anxiety disorders: A meta-analysis of 30 years of research. J. Affect. Disord..

[40] Florek S., Pudlo R., Gościniewicz P., Mrukwa-Kominek E. (2023). Mental disorders in people with keratoconus. Curr. Probl. Psychiatry.

[41] Giedd K.K., Mannis M.J., Mitchell G.L., Zadnik K. (2005). Personality in keratoconus in a sample of patients derived from the internet. Cornea.

[42] Mannis M.J., Ling J.J., Kyrillos R., Barnett M. (2018). Keratoconus and Personality—A Review. Cornea.

[43] Cooke C.A., Cooper C., Dowds E., Frazer D.G., Jackson A.J. (2003). Keratoconus, myopia, and personality. Cornea.

[44] Mannis M.J., Morrison T.L., Zadnik K., Holland E.J., Krachmer J.H. (1987). Personality trends in keratoconus. An. Anal. Arch. Ophthalmol..

[45] Aslan M.G., Besenek M., Akgoz H., Satılmaz M.F., Hocaoglu C. (2021). Evaluation of Personality Features and Mental State of Keratoconus Patients. Beyoglu Eye J..

[46] Korzeniowski L., Pużyński S., Cwynar S. (1986). Encyklopedyczny Słownik Psychiatrii.

[47] Maurice-Stam H., Nijhof S.L., Monninkhof A.S., Heymans H.S.A., Grootenhuis M.A. (2019). Review about the impact of growing up with a chronic disease showed delays achieving psychosocial milestones. Acta Paediatr..

[48] Al-Dairi W., Al Sowayigh O.M., Al Saeed A.A., Alsaad A. (2020). Depression among Keratoconus Patients in Saudi Arabia. Cureus.

[49] Yildiz M., Turhan S.A., Yargı B., Ergün S., Örnek E., Baz F., Toker A.E. (2021). Psychiatric morbidity of patients with keratoconus: A cross-sectional study. J. Psychosom. Res..

[50] Pudlo R., Jaworska I., Fuczyło K., Jarema M. (2021). Psychiatryczne aspekty transplantacji serca In Diagnozowanie i Leczenie Zaburzeń Psychicznych w Schorzeniach Somatycznych.

