# Supplementary material for: Psychological and Psychiatric Characteristics of People with Keratoconus
Source: Reports (MDPI). 2024 Aug 3;7(3):67. doi: 10.3390/reports7030067 (PMC12225485; doi:10.3390/reports7030067)
Supplement: Supplementary file 1 [file reports-07-00067-s001.zip › reports-3079495-supplementary.pdf]

Supplementary Material

Table S1. Descriptive statistics and results of Shapiro-Wilk tests among analysed variables.

Variable	Mean	SD	*N*	Min	Max	W	*p*
**Whole study population**							
• HDRS	3.586	4.415		0.000	21.000	0.793	*<0.001
• BDI	5.272	6.882		0.000	33.000	0.765	*<0.001
• STAI X-1	35.133	7.743		23.000	62.000	0.922	*<0.001
• STAI X-2	37.333	8.592		23.000	63.000	0.945	*<0.01
• paranoid personality	5.394	4.451		0.000	18.000	0.923	*<0.001
• schizoid personality	5.182	4.514		0.000	20.000	0.874	*<0.001
• schizotypal personality	4.030	3.558	99	0.000	16.000	0.904	*<0.001
• antisocial personality	3.586	3.648		0.000	17.000	0.828	*<0.001
• borderline personality	3.626	3.968		0.000	20.000	0.8410.	*<0.001*<0
• histrionic personality	4.323	3.920		0.000	18.000	896	.001
• narcissistic personality	3.576	3.747		0.000	16.000	0.862	*<0.001
• avoidant personality	4.737	3.869		0.000	17.000	0.920	*<0.001
• dependent personality	6.727	4.460		0.000	18.000	0.958	*<0.01
• obsessive-compulsive personality	8.283	4.366		0.000	20.000	0.978	0.094
**Men from study population**							
• HDRS	3.151	4.088		0.000	20.000	0.777	*<0.001
• BDI	4.918	6.055		0.000	32.000	0.767	*<0.001
• STAI X-1	33.604	7.204		23.000	61.000	0.924	*<0.01
• STAI X-2	35.811	8.528		23.000	63.000	0.915	*<0.01
• paranoid personality	5.356	4.620		0.000	18.000	0.912	*<0.001
• schizoid personality	4.863	4.114		0.000	18.000	0.877	*<0.001
• schizotypal personality	3.808	3.623	73	0.000	16.000	0.885	*<0.001
• antisocial personality	4.027	3.989		0.000	17.000	0.847	*<0.001
• borderline personality	3.384	3.900		0.000	15.000	0.8270.	*<0.001*<0
• histrionic personality	4.260	3.916		0.000	16.000	895	.001
• narcissistic personality	3.699	3.922		0.000	13.000	0.852	*<0.001
• avoidant personality	4.466	3.701		0.000	17.000	0.908	*<0.001
• dependent personality	6.205	4.598		0.000	18.000	0.929	*<0.001
• obsessive-compulsive personality	8.178	4.408		0.000	19.000	0.977	0.215
**Women from study population**							
• HDRS	4.808	5.115		0.000	21.000	0.838	*<0.001
• BDI	8.000	8.532		0.000	33.000	0.785	*<0.001
• STAI X-1	38.818	7.914		29.000	62.000	0.874	*<0.01
• STAI X-2	41.000	7.752		30.000	56.000	0.935	0.160
• paranoid personality	5.500	4.022		0.000	16.000	0.929	0.072
• schizoid personality	6.077	5.477		0.000	20.000	0.874	*<0.01
• schizotypal personality	4.654	3.358	26	0.000	12.000	0.9290.	0.074
• antisocial personality	2.346	2.038		0.000	7.000	884	*<0.01
• borderline personality	4.308	4.155		0.000	20.000	0.7900.	*<0.001*<0
• histrionic personality	4.500	4.002		0.000	18.000	970	.01
• narcissistic personality	3.231	3.253		0.000	16.000	0.762	*<0.001
• avoidant personality	5.500	4.292		0.000	16.000	0.9430.	0.161
• dependent personality	8.192	3.753		0.000	16.000	9800.95	0.881
• obsessive-compulsive personality	8.577	4.319		0.000	20.000	9	0.365
**Whole control group**							
• HDRS	3.478	4.775		0.000	20.000	0.752	*<0.001
• BDI	5.967	6.336		0.000	27.000	0.844	*<0.001
• STAI X-1	36.141	8.590	92	23.000	61.000	0.951	*<0.01
• STAI X-2	38.239	8.477		23.000	56.000	0.968	*<0.05
• paranoid personality	3.957	3.328		0.000	12.000	0.914	*<0.001
• schizoid personality	5.098	3.632		0.000	17.000	0.922	*<0.001
• schizotypal personality	2.707	2.612		0.000	12.000	0.8530.	*<0.001*<0

• antisocial personality	2.261	2.178		0.000	9.000	866	.001
• borderline personality	3.446	3.300		0.000	12.000	0.8820.	*<0.001*<0
• histrionic personality	4.022	3.078		0.000	14.000	932	.001
• narcissistic personality	3.815	3.214		0.000	15.000	0.897	*<0.001
• avoidant personality	4.978	3.947		0.000	16.000	0.898	*<0.001
• dependent personality	6.511	3.810		0.000	19.000	0.941	*<0.001
• obsessive-compulsive personality	7.587	4.305		1.000	20.000	0.963	*0.01
**Men from control group**							
• HDRS	2.889	3.946		0.000	16.000	0.751	*<0.001
• BDI	5.222	5.920		0.000	23.000	0.828	*<0.001
• STAI X-1	35.389	8.455		23.000	59.000	0.952	*<0.05
• STAI X-2	36.741	8.579		23.000	55.000	0.948	*<0.05
• paranoid personality	4.278	3.212		0.000	12.000	0.936	*<0.01
• schizoid personality	5.796	4.002		0.000	17.000	0.940	*<0.01
• schizotypal personality	2.981	2.858	54	0.000	12.000	0.843	*<0.001
• antisocial personality	2.759	2.457		0.000	9.000	0.894	*<0.001
• borderline personality	3.093	2.980		0.000	12.000	0.8880.	*<0.001*<0
• histrionic personality	4.019	2.956		0.000	10.000	929	.01
• narcissistic personality	4.519	3.463		0.000	15.000	0.926	*<0.01
• avoidant personality	4.648	3.812		0.000	16.000	0.886	*<0.001
• dependent personality	6.019	3.207		0.000	15.000	0.950	*<0.05
• obsessive-compulsive personality	8.463	4.500		1.000	20.000	0.971	0.207
**Women from control group**							
• HDRS	4.316	5.705		0.000	20.000	0.767	*<0.001
• BDI	7.026	6.824		0.000	27.000	0.852	*<0.001
• STAI X-1	37.211	8.780		23.000	61.000	0.948	0.076
• STAI X-2	40.368	7.961		26.000	56.000	0.973	0.483
• paranoid personality	3.500	3.478		0.000	12.000	0.866	*<0.001
• schizoid personality	4.105	2.788		0.000	12.000	0.913	*<0.01
• schizotypal personality	2.316	2.195	38	0.000	8.000	0.8770.	*<0.001*<0
• antisocial personality	1.553	1.465		0.000	6.000	878	.001
• borderline personality	3.947	3.690		0.000	12.000	0.8790.	*<0.001*<0
• histrionic personality	4.026	3.284		0.000	14.000	907	.01
• narcissistic personality	2.816	2.545		0.000	12.000	0.859	*<0.001
• avoidant personality	5.447	4.137		0.000	16.000	0.907	*<0.01
• dependent personality	7.211	4.485		0.000	19.000	0.941	*<0.05
obsessive-compulsive personality	6.342	3.722		1.000	16.000	0.943	0.053

Table S2. Spearman rank correlations within the women from the study population.

Variable	HDRS	BDI	STAI X-1	STAI X-2
HDRS	1.000	0.663 *	0.616 *	0.579 *
BDI		1.000	0.511 *	0.811 *
STAI X-1			1.000	0.517 *
STAI X-2				1.000
paranoid personality	0.331	0.549 *	0.590 *	0.584 *
schizoid personality	0.364	0.555 *	0.472 *	0.439 *
schizotypal personality	0.395 *	0.467 *	0.302	0.380
antisocial personality	0.188	0.276	0.026	0.091
borderline personality	0.246	0.504 *	0.476 *	0.629 *
histrionic personality	0.262	0.495 *	0.235	0.450 *
narcissistic personality	0.187	0.317	0.128	0.172
avoidant personality	0.428 *	0.680 *	0.513 *	0.709 *
dependent personality	0.350	0.516 *	0.446 *	0.466 *
obsessive-compulsive personality	0.189	0.425 *	0.085	0.212

*-statistically significant at $p < 0.05$; HDRS – Hamilton Depressing Rating Scale; BDI - Beck's Depression Inventory; STAI X-1 - State-Trait Anxiety Inventory for anxiety as a condition [ten]; STAI X-2 - State-Trait Anxiety Inventory for anxiety as a trait [ten].

Table S3. Spearman rank correlations within the men from the study population.

Variable	HDRS	BDI	STAI X-1	STAI X-2
HDRS	1.000	0.440 *	0.195	0.404 *
BDI		1.000	0.364 *	0.507 *
STAI X-1			1.000	0.517 *
STAI X-2				1.000
paranoid personality	0.432 *	0.659 *	0.462 *	0.547 *
schizoid personality	0.399 *	0.391 *	0.428 *	0.470 *
schizotypal personality	0.395 *	0.723 *	0.429 *	0.511 *
antisocial personality	0.415 *	0.541 *	0.363	0.294
borderline personality	0.433 *	0.708 *	0.426 *	0.658 *
histrionic personality	0.374 *	0.596 *	0.272 *	0.437 *
narcissistic personality	0.466 *	0.527 *	0.208	0.468 *
avoidant personality	0.395 *	0.598 *	0.314 *	0.620 *
dependent personality	0.343 *	0.522 *	0.410 *	0.711 *
obsessive-compulsive personality	0.264 *	0.504 *	0.264	0.410 *

*-statistically significant at $p < 0.05$; HDRS – Hamilton Depressing Rating Scale; BDI - Beck's Depression Inventory; STAI X-1 - State-Trait Anxiety Inventory for anxiety as a condition [ten]; STAI X-2 - State-Trait Anxiety Inventory for anxiety as a trait [ten];.

Table S4. Spearman rank correlations within the women of the control group.

Variable	HDRS	BDI	STAI X-1	STAI X-2
HDRS	1.000	0.721 *	0.522 *	0.629 *
BDI		1.000	0.535 *	0.767 *
STAI X-1			1.000	0.667 *
STAI X-2				1.000
paranoid personality	0.503*	0.599 *	0.373 *	0.474 *
schizoid personality	0.243	0.341 *	0.306	0.389 *
schizotypal personality	0.543*	0.599 *	0.278	0.500 *
antisocial personality	−0.080	−0.156	−0.330 *	−0.301
borderline personality	0.635*	0.732 *	0.424 *	0.665 *
histrionic personality	0.458*	0.530 *	0.231	0.364 *
narcissistic personality	0.480*	0.571 *	0.360 *	0.487 *
avoidant personality	0.367*	0.457 *	0.165	0.617 *
dependent personality	0.348*	0.519 *	0.292	0.580 *
obsessive-compulsive personality	0.354*	0.213	0.148	0.159

*-statistically significant at $p < 0.05$; HDRS – Hamilton Depressing Rating Scale; BDI - Beck's Depression Inventory; STAI X-1 - State-Trait Anxiety Inventory for anxiety as a condition [ten]; STAI X-2 - State-Trait Anxiety Inventory for anxiety as a trait [ten];.

Table S5. Spearman rank correlations within the men of the control group.

Variable	HDRS	BDI	STAI X-1	STAI X-2
HDRS	1.000	0.692 *	0.642 *	0.590 *
BDI		1.000	0.756 *	0.669 *
STAI X-1			1.000	0.712 *
STAI X-2				1.000
paranoid personality	0.449 *	0.584 *	0.525 *	0.438 *
schizoid personality	0.287 *	0.630 *	0.588 *	0.388 *

schizotypal personality	0.338 *	0.543 *	0.384 *	0.379 *
antisocial personality	0.112	0.022	0.018	−0.150
borderline personality	0.524 *	0.634 *	0.675 *	0.665 *
histrionic personality	0.098	0.269 *	0.114	0.151
narcissistic personality	−0.133	0.058	0.000	0.009
avoidant personality		0.596 *	0.530 *	0.625 *
dependent personality	0.474 *	0.575 *	0.413 *	0.517 *
obsessive-compulsive personality	0.162	0.408 *	0.383 *	0.426 *

*-statistically significant at $p < 0.05$; HDRS – Hamilton Depressing Rating Scale; BDI - Beck's Depression Inventory; STAI X-1 - State-Trait Anxiety Inventory for anxiety as a condition [ten]; STAI X-2 - State-Trait Anxiety Inventory for anxiety as a trait [ten].

Table S6. Comparison of study parameters among men between test and control group (Mann-Whitney U test, $p < 0.05$).

Variable	Study Group (n = 73)			Control Group (n = 54)			Cohen's d	Z	p
	Average	SD	Median	Average	SD	Median			
HDRS	3.151	4.088	1.000	2.889	3.946	1.000	0.082	0.458	0.647
BDI	4.918	6.055	3.000	5.222	5.920	3.000	0.049	−0.276	0.783
STAI X-1	4.415	1.896	4.000	4.944	2.141	5.000	0.246	−1.262	0.207
STAI X-2	4.019	2.374	4.000	4.611	2.573	4.000	0.219	−1.125	0.261
paranoid personality	4.274	2.440	4.000	3.796	1.752	4.000	0.136	0.761	0.377
schizoid personality	4.444	2.349	4.000	4.926	2.171	5.000	0.286	−1.476	0.114
schizotypal personality	4.438	2.444	4.000	4.056	2.060	4.000	0.164	0.919	0.358
antisocial personality	4.534	2.334	4.000	3.833	1.799	4.000	0.249	1.387	0.165
borderline personality	3.877	2.566	4.000	3.815	2.129	4.000	0.032	−0.180	0.857
histrionic personality	4.534	2.489	4.000	4.500	1.881	4.500	0.030	−0.168	0.866
narcissistic personality	4.014	2.579	4.000	4.833	1.979	5.000	0.347	−1.921	0.055
avoidant personality	4.781	2.335	4.000	4.963	2.119	4.500	0.055	−0.310	0.757
dependent personality	5.260	2.571	5.000	5.167	1.830	5.000	0.053	−0.295	0.768
obsessive-compulsive personality	4.918	2.247	5.000	4.981	2.319	5.000	0.035	-0.193	0.847

HDRS – Hamilton Depressing Rating Scale; BDI - Beck's Depression Inventory; STAI X-1 - State-Trait Anxiety Inventory for anxiety as a condition [ten]; STAI X-2 - State-Trait Anxiety Inventory for anxiety as a trait [ten]; SD – standard deviation.

Table S7. Comparative analysis of the severity of depressive and anxiety symptoms in the test and control group (chi-square test, $p < 0.05$).

Variable	Study Group		Control Group		Chi-quadrat	p
	N	Percentage [%]	N	Percentage [%]		
BDI						
• absence of depression (0–11)	85	85.86	79	85.87	6.697	0.082
• mild depression (12–19)	9	09.09	8	08.70		
• moderate depression (20–25)	0	00.00	4	04.35		
• severe depression (>26)	5	05.05	1	01.09		
HDRS						
• absence of depression (0–6)	79	79.80	73	79.35	0.266	0.966
• mild depression (7–11)	15	15.15	13	14.13		
• moderate depression (13–17)	3	03.03	4	04.35		
• severe depression (18–29)	2	02.02	2	02.17		
• very sever depression (>30)	0	00.00	0	00.00		
STAI X-1						
• <7	59	78.67	67	72.83	0.761	0.383
• ≥7	16	21.33	25	27.17		
STAI X-2					0.588	0.443

• <7	60	80.00	69	75.00		
• ≥7	15	20.00	23	25.00		

N – number of people.

Table S8. Comparative analysis of the severity of depressive and anxiety symptoms among women in the test and control group (chi-square test, $p < 0.05$).

Variable	Study group		Control group		Chi-quadrat	*p*
	N	Percentage [%]	*N*	Percentage [%]		
BDI						
• absence of depression (0–11)	22	84.62	32	84.21		
• mild depression (12–19)	1	03.85	3	07.89	3.733	0.292
• moderate depression (20–25)	0	00.00	2	05.26		
• severe depression (>26)	3	11.54	1	02.63		
HDRS						
• absence of depression (0–6)	19	73.08	29	76.32		
• mild depression (7–11)	4	15.38	4	10.53		
• moderate depression (13–17)	2	07.69	3	07.89	0.380	0.944
• severe depression (18–29)	1	03.85	2	05.26		
• very sever depression (>30)	0	00.00	0	00.00		
STAI X-1						
• <7	15	68.18	28	73.68	0.208	0.649
• ≥7	7	31.82	10	26.32		
STAI X-2						
• <7	16	72.73	29	76.32	0.096	0.757
• ≥7	6	27.27	9	23.68		

N – number of people.

Table S9. Comparative analysis of the severity of depressive and anxiety symptoms among men in the test and control group (chi-square test, $p < 0.05$).

Variable	Study Group		Control Group		Chi-quadrat	*p*
	N	Percentage [%]	*N*	Percentage [%]		
BDI						
• absence of depression (0–11)	63	86.30	47	87.04		
• mild depression (12–19)	8	10.96	5	09.26	4.273	0.233
• moderate depression (20–25)	0	00.00	2	03.70		
• severe depression (>26)	2	02.74	0	00.00		
HDRS						
• absence of depression (0–6)	60	82.19	44	81.48		
• mild depression (7–11)	11	15.07	9	16.67		
• moderate depression (13–17)	1	01.37	1	01.85	0.838	0.840
• severe depression (18–29)	1	01.37	0	00.00		
• very sever depression (>30)	0	00.00	0	00.00		
STAI X-1						
• <7	44	83.02	39	72.22	1.792	0.181
• ≥7	9	16.98	15	27.78		
STAI X-2						
• <7	44	83.02	40	74.07	1.268	0.260
• ≥7	9	16.98	14	25.93		

N – number of people.
